# Increasing Abnormal Involuntary Movement Scale (AIMS) Screening for Tardive Dyskinesia in an Outpatient Psychiatry Clinic: A Resident-Led Outpatient Lean Six Sigma Initiative

**DOI:** 10.7759/cureus.39486

**Published:** 2023-05-25

**Authors:** Arindam C Chakrabarty, Jeffrey I Bennett, Talha J Baloch, Rohit P Shah, Cassie Hawk, Tyler Natof

**Affiliations:** 1 Psychiatry, Southern Illinois University School of Medicine, Springfield, USA; 2 Psychiatry, Comprehensive Psych Solutions, Chicago, USA; 3 Quality and Safety, Decatur Memorial Hospital, Decatur, USA

**Keywords:** medication side-effects, psychotropic medications, serious mental illness, resident education, quality improvement, lean six sigma, aims screening, tardive dyskinesia

## Abstract

Objective

To increase compliance with Abnormal Involuntary Movement Score (AIMS) documentation for patients taking antipsychotics to recognize and treat tardive dyskinesia in the psychiatry outpatient clinic.

Methods

The Lean Six Sigma quality improvement (QI) model, utilizing DMAIC steps of define, measure, analyze, improve, control, was followed. Psychiatry attendings and residents were surveyed to assess reasons for AIMS non-documentation, and they ranked their preferred solutions to increase compliance. A random sample of patient charts for individuals on antipsychotic medications was obtained to determine AIMS documentation compliance prior to and following the implementation of improvements.

Results

The most highly ranked solution was implementing a one-hour AIMS training session. Three months post-intervention, a random sample of 60 patient charts showed that 87% (52/60) of patients had AIMS documented which was a significant increase compared to 3% (1/30) pre-intervention (p<0.001).

Conclusion

An annual, one-hour AIMS training session for residents improved rates of AIMS documentation.

## Introduction

Tardive dyskinesia (TD) is a syndrome characterized by abnormal involuntary movements of the patient's face, mouth, trunk, or limbs, which affects 20%-30% of patients who have been treated for months or years with neuroleptic medications [1,2]. The movements of the patient's limbs and trunk are sometimes called choreoathetoid movements, which mean a dance-like movement that repeats itself and has no rhythm. The Abnormal Involuntary Movement Scale (AIMS) is used not only to detect tardive dyskinesia but also to follow the severity of a patient's TD over time [3]. It is a valuable tool for clinicians who are monitoring the effects of long-term treatment with neuroleptic medications and for researchers studying the effects of these drugs. The AIMS is administered every three to six months to monitor the patient for the development of TD. For most patients, TD develops three months after the initiation of neuroleptic therapy. In elderly patients, however, TD can develop after as little as one month. Pharmacological treatment options were recently approved by the FDA for the treatment of tardive dyskinesia [3-5].

Between October 2016 and September 2017, only one patient seen at the clinic prescribed an antipsychotic medication had an AIMS score documented in electronic health record (EHR) in the NoteForms, Flowsheets, or order sections. There was a consideration that this was potentially being documented as free text. This may have been related to recent changes in EHR, clinic location, and personnel changes. The AIMS is a well-known 12-item, validated scale to assess TD. Items 1-7 assess involuntary movements across body regions, with a score ranging from zero (no dyskinesia) to four (severe, maximal amplitude, and persistence during observation of abnormal movements). Items 8-12 assess global judgements and dental status [6].

The American Psychiatric Task Force report on tardive dyskinesia in its guidelines for the avoidance and management of TD, recommended regular examinations for early signs of choreoathetosis and oral-lingual dyskinesias [3]. Ideally, examinations that use instruments such as the AIMS should be done before the institution of neuroleptic drug therapy and then repeated on a regular basis [3]. Previous American Psychiatric Association (APA) practice guidelines stated that patients receiving conventional antipsychotic medications should be monitored for TD every three to four months, and patients on newer atypical antipsychotics should be monitored for TD every five to six months [7]. Updated 2020 APA guidelines stipulate that patients with schizophrenia should be assessed with AIMS every six months if they are high-risk for TD and every 12 months otherwise [8].

Two vesicular monoamine transporter-2 (VMAT-2) inhibitors, valbenazine and deutetrabenazine, are effective in treating TD, both acutely and long-term [4-5]. They were FDA-approved in 2017 for TD treatment [4,5,9]. This current study does not investigate these pharmaceuticals per se. However, the development of these pharmaceuticals underscores the importance of this study’s goal: increasing compliance with AIMS and AIMS documentation to increase TD detection and monitoring.

## Materials and methods

Objectives

A large number of patients seen in the psychiatry clinic are on medications that may cause tardive dyskinesia. AIMS should ideally be administered and documented to each patient on these medications at least every six months. However, anecdotal reports from clinic staff indicate that this may not always be the case. The objectives were to: (a) assess the frequency with which AIMS assessments are being done for patients on neuroleptic medications, (b) identify the reasons for failure to assess, and (c) develop a process to increase the compliance with AIMS assessment and documentation. Regular assessment of AIMS will help detection and early identification of the disease, ensure treatment and follow-up, and generate data on the long-term effects of these drugs.

Protection of human subjects

There was minimal risk to patients, as this is a limited chart review. Access to the patient’s personal information is necessary in order to gather the identified data, as would be consistent in the course of regular practice. No protected health information (PHI) was collected. The de-identified data is only available to the principal investigator and co-investigators and was stored in a locked cabinet within the Department. Survey results are reported in the aggregate. The surveys are anonymous. Institutional Review Board waiver was obtained.

Pre-intervention data

An informal poll of providers (n=5) in the psychiatry outpatient clinic revealed that AIMS assessments may not get done every six months for patients on neuroleptic medications. In addition, a random sample of 30 patient charts of adult patients on antipsychotic medications from the psychiatry outpatient clinic were reviewed to verify the results of the informal poll. Only one patient had documentation of AIMS. The randomly selected patient charts were dated between October 2016 and September 2017 and were thoroughly evaluated to see if an AIMS assessment was performed or not.

**Figure 1:**
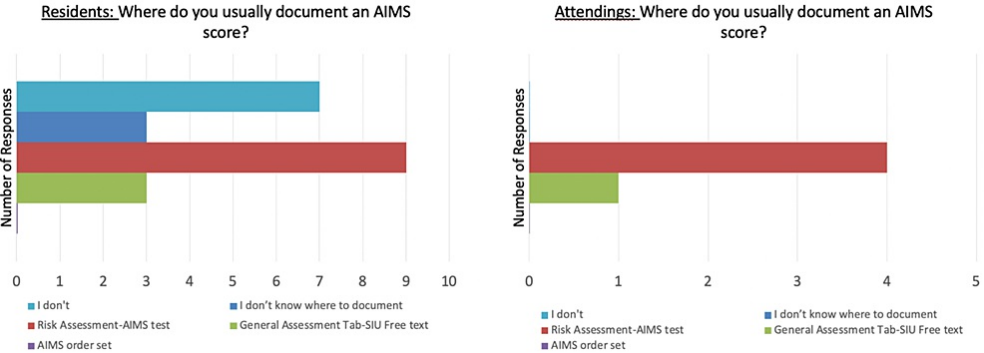
Resident/attending responses to AIMS documentation. AIMS: Abnormal Involuntary Movement Score

**Figure 2:**
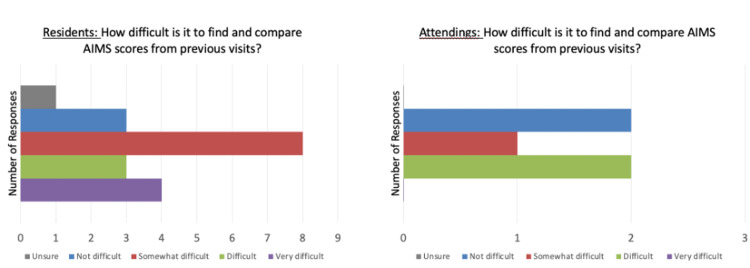
Resident/attending responses to difficulty with longitudinal comparison of AIMS score. AIMS: Abnormal Involuntary Movement Score

Through the Lean Six Sigma process, the team identified several possible causes for this problem. Documentation takes time and there are time limits for appointments. Providers may be conducting the AIMS screening, but it is not being documented in a manner that is easy to access such as, in the free text section of the patient’s chart (Figure 1). In the current EHR, it is difficult to find and compare previous AIMS scores to facilitate clinical intervention (Figure 2). Further, providers may not know how to use the tool or even be aware that the tool exists. One potential explanation is the switch to a new EHR that does not include automated alerts to physicians to conduct and document an AIMS result.

Intervention

**Figure 3:**
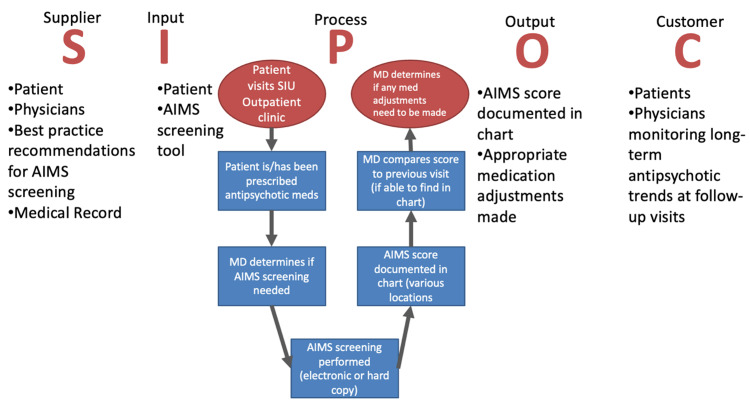
SIPOC (Suppliers, Input, Process steps, Output, and Customers) analysis generated by the authors in preliminary study design. Authors' own creation

To facilitate AIMS training, all residents (n=26) and attendings (n=5) were surveyed regarding their experience and knowledge of the AIMS tool. The survey included questions related to whether the provider uses the AIMS tool, how often the AIMS should be completed, whether the respondent has been trained to use the AIMS, and what prevents the respondent from using the AIMS (Figure 2). The results of this survey informed the development of an AIMS curriculum for the Department of Psychiatry. Additionally, other interventions like changes to the EHR documentation process, and office policy may be required to be effective. These may include measures like reducing the number of steps required to capture data, tracking the dates of last completed AIMS, standardizing the frequency of AIMS administration, and providing an alert/reminder to providers. A SIPOC (Suppliers, Input, Process steps, Output, and Customers) tool was used to map the process important to the AIMS screening process (Figure 3).

## Results

The Lean Six Sigma quality improvement (QI) model, utilizing DMAIC steps of Define, Measure, Analyze, Improve, Control, was followed.

Define

The goal of this project is to increase compliance with AIMS screening of patients seen in the outpatient psychiatry clinic who are prescribed an antipsychotic medication.

Measure

**Figure 4:**
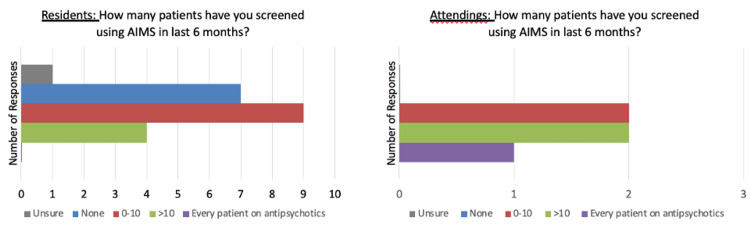
Resident/attending responses to AIMS usage. AIMS: Abnormal Involuntary Movement Score

As stated above, between October 2016 and September 2017, in a random sample of 30 patients, one patient seen at this study’s outpatient psychiatry clinic prescribed an antipsychotic medication had an AIMS score documented in a readily searchable portion of the chart. A third of the residents surveyed reported that they had screened patients on antipsychotic medication with AIMS. One of the five attendings surveyed indicated they screen every patient on an antipsychotic with AIMS (Figure 4).

Analyze

**Figure 5:**
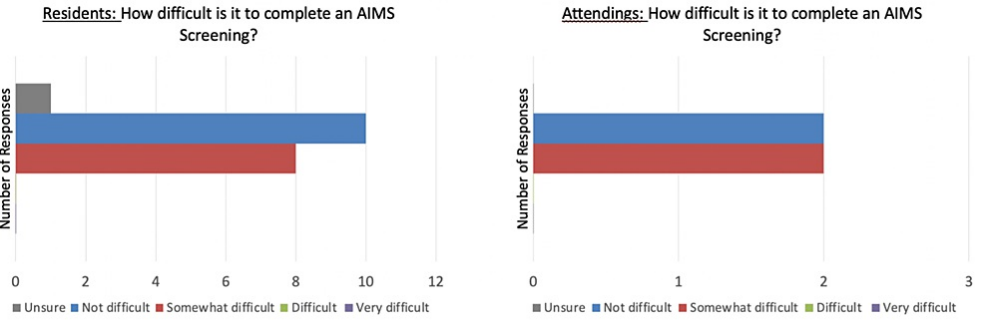
Resident/attending responses to difficulty of AIMS screening. AIMS: Abnormal Involuntary Movement Score

**Figure 6:**
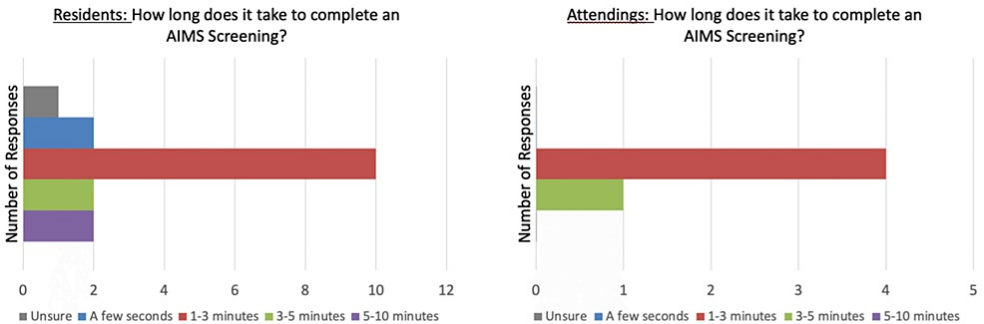
Resident/attending responses to time for AIMS documentation. AIMS: Abnormal Involuntary Movement Score

One hypothesis for poor AIMS documentation is the potential difficulty in administration of the screening. Residents and attendings were surveyed on a variety of questions to determine potential obstacles. Notably, they were allowed to mark multiple responses. None of the residents or attendings indicated that completing an AIMS screening is “difficult” or “very difficult” (Figure 5). Another hypothesis was that completing the AIMS screening is too time-consuming. The mode response (10 of 17 residents and 4 of 5 attendings) when surveyed “How long does it take to complete an AIMS screening” was “1-3 minutes” (Figure 6). When the survey question was directly posed, “What prevents you from using the AIMS screening tool,” the most common response (11 of 31 responses) was “no formal training” followed by “I’m not reminded to” (8 of 31) (Table 1).

**Table 1:** Resident responses when surveyed on reasons for AIMS non-compliance AIMS: Abnormal Involuntary Movement Score

	Number of Resident Responses (n=31)	Percent of Total Responses
No formal training	11	35.484
I'm not reminded to	8	25.806
Unclear how often patients should be screened	5	16.129
Extra Clicks	4	12.903
Time constraint	3	9.677
Unclear where to document the score	0	0.000
I screen every patient	0	0.000
Total Responses	31	100

Improve

**Figure 7:**
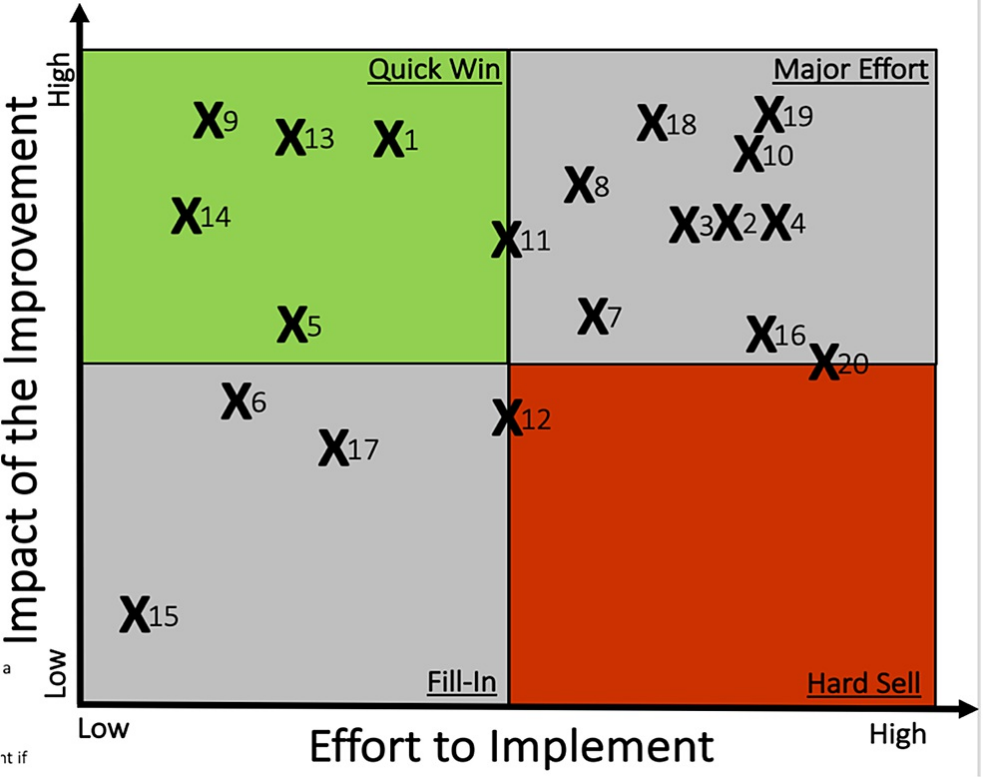
Feasibility of Potential Interventions. Specific means of implementing 20 different AIMS training were scored based on their effort to implement (x-axis) and impact of the improvement (y-axis). Based on the quadrants of the plot, interventions were assigned to one of four categories: Low effort/high impact, low effort/low impact, high effort/low impact, and high effort impact. X1 – One-hour session and video education X2 – Incorporate training in first-year lectures X3 – Include training as part of new trainee orientation X4 – Include in orientation at the beginning of each year/rotation X5 – PowerPoint training available on department computer drive with completion tracking X6 – PowerPoint training on department computer drive as a refresher X7 – Noon conference topic in July X8 – Include training as a CBL and track completion rate X9 – Reminders given in monthly clinic operation meetings X10 – Providers enter an order for AIMS to track score X11 – Edit documentation software so AIMS score can be automatically tracked without the need for order X12 – AIMS assessment auto-populates a scores X13 – Standardize/train attending’s expectations from residents X14 – Watch YouTube video of example patient X15 – Put up an educational poster in the clinic space X16 – Make a standardized patient video X17 – Send out AIMS completion scores every 6 months for accountability X18 – Nurse gives a paper copy of the AIMS tool to a physician for every encounter on individual on antipsychotic medications X19 – Complete an AIMS assessment at every patient encounter X20 – Pre-check chart on day prior to appointment to determine the need for AIMS screening

Six interventions (notated as 1, 5, 9, 11, 13, and 14 in Figure 7) were identified as low effort and high impact, so-called “quick wins.” Three of these involved physician training: watching a YouTube video of an example patient undergoing AIMS screening, conducting a one-hour session including a video explaining how to complete AIMS and where it should be documented in the EHR, and providing PowerPoint training on AIMS in addition to tracking who had completed AIMS documentation. Two of the six involved providing reminders, either in the form of monthly reminders in clinic operations meetings or as part of resident training given by attendings. The last of the six focused on the ability to track AIMS without entering this as an order. Five other interventions that either had a high impact or a reasonable level of effort to impact benefit were included to formulate another survey.

Study results

**Figure 8:**
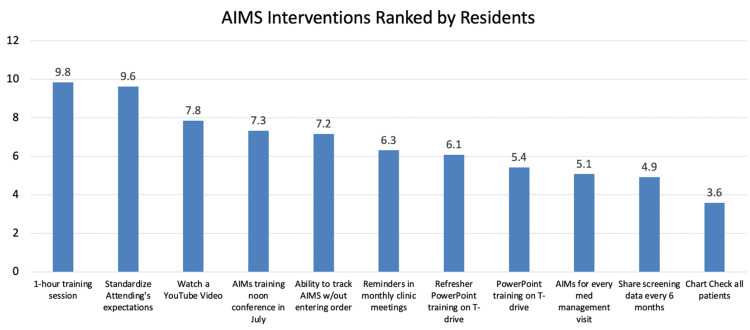
Proposed interventions ranked by residents. AIMS: Abnormal Involuntary Movement Score

The survey found that a one-hour training session was the most highly rated intervention (Figure 8). Based on the results of the surveys and focus group discussions, a one-hour training session was implemented by the study team for AIMS training. No specific training for attendings or other staff was implemented. Follow-up data review was planned at three months and six months post-intervention.

**Figure 9:**
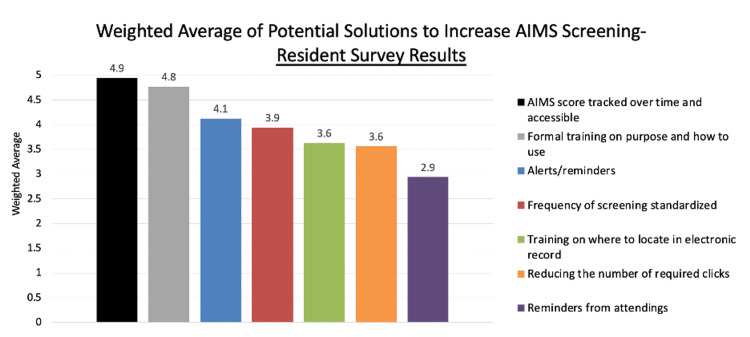
Weighted average of potential solutions. AIMS: Abnormal Involuntary Movement Score

When asked, “What prevents you from using the AIMS Screening Tool?” among residents surveyed, the most common response was “no formal training,” (Table 1). These data suggested that formal training was the single most popular option among residents. With the broad goal of implementing formal training, more specific choices were then evaluated. Nineteen different options were scored on two different metrics: the effort to implement, and the impact of improvement. An ideal solution would be low-effort but high-impact. The results of this scoring are shown in Figure 7. The option with the lowest effort and highest impact was “reminders given in a monthly clinic operations meeting.” Additionally, residents ranked potential solutions to increase AIMS screening, and weighted averages were calculated for each solution based on focus group discussions (See Figure 9 for details).

Three-month follow-up (data collected in February 2020) showed that 52 out of 60 (87%) random charts had AIMS documented. This showed a significant improvement from a 3% documentation rate pre-intervention (p<0.001). Six-month data originally set to be collected in May 2020 was not collected due to the COVID-19 pandemic disrupting clinic flows, tele-health appointments, and deployment of resources to combat the pandemic.

## Discussion

This QI project was an initiative led by third-year psychiatry residents with a hospital-based Lean Six Sigma black belt mentor. It addressed a deficiency that residents had noted in AIMS documentation with changes in the department and the EHR. The increase in AIMS documentation from 3% to 87% (p<0.001) shows the effectiveness of the educational intervention. The aim of the QI project was to achieve a 100% documentation rate. A total of 87% documentation rate may reflect the need for additional changes to the process. However, it may also be explained by three months of data collection, where AIMS testing is recommended once every 6-12 months. Further longitudinal follow-up data would provide information on continued efficacy. Given the high risk of developing tardive dyskinesia following the use of psychiatric medications and the recent approval for medications to treat tardive dyskinesia, this is an important intervention [1-5].

Similar improvements in AIMS screening were reported by other researchers. The Tardive Dyskinesia Assessment Working Group suggests that the AIMS is a valid assessment tool for TD both for research and clinical practice but does not report data on improvement in screening rates. A 2019 study reported an increase in AIMS implementation from 0% to 80% in 12 weeks in an outpatient private practice when the scale was administered by a Doctor of Nursing Practice (DNP) student during each patient visit [10]. However, the goal of the study was to identify TD and involved a single researcher administering AIMS to each patient to study improvement in patient outcomes and used the Plan-Do-Study-Act QI model. A 2021 QI study suggested an 85.1% increase in TD screening for high-risk patients. This was a study done in the inpatient setting with daily screening by a pharmacist for high-risk individuals [11]. Another study showed that using the model for improvement showed improvement in screening for abnormal involuntary movements using AIMS after eight weeks [12].

Our study is the first study we could find that aims to increase the awareness and use of screening instruments for TD in resident education. This project shows that resident-led QI initiatives can produce significant changes. It also provides residents with an opportunity to learn about designing and implementing Lean Six Sigma principles and quality improvement in training. The annual one-hour AIMS training will continue, and long-term data assessing AIMS documentation will be tracked. Other highly-ranked interventions by residents, such as efforts to standardize attendings’ expectations, will be tested and compared to each other.

In analyzing potential reasons for poor AIMS documentation rate, time was a potential factor. Residents and attendings were surveyed on the length of time to complete an AIMS screening; however, responses were not contextualized based on the average length of a patient encounter for the respondents (i.e., “1-3 minutes” is proportionally a much greater amount of time and therefore imposes a greater burden on AIMS screening for a 10-minute patient encounter than a 30-minute patient encounter). Another consideration of our data is the heterogeneity of survey responses with respective questions: the total number of responses varies from question to question. This is because respondents were allowed to choose more than one response but did not always do so. However, allowing residents and attendings to indicate multiple survey responses captured a broader range of input regarding perceived reasons for difficulties in conducting and documenting AIMS screening.

The study shows that changes in electronic health records, time constraints, and limited exposure to TD screening education can be overcome with continuing education efforts. The results of increased AIMS screening show the value of near-peer teaching and learning and it has implications for resident teaching and education. Residents are aware of the challenges presented by the system and are uniquely placed to design appropriate interventions. The study demonstrates that a simple one-hour resident intervention can significantly increase AIMS implementation and screening for tardive dyskinesia. This has high clinical importance as it allows for earlier diagnosis and management of TD especially in light of recent advances in therapeutics for TD improving quality of care.

## Conclusions

A resident-led QI project using the Lean Six Sigma model in the outpatient clinic showed a significant increase in rates of AIMS screening in patients receiving psychotropic medications. A one-hour educational intervention increased the rates of screening from 3% to 87%. This is an important intervention in view of the nature of the side effects, its prevalence, and the recent approval of effective medications for treatment. The study also sets the stage for future interventions designed by residents to improve the quality of patient care as well as provider education.
